# Median effective dose of esketamine for intranasal premedication in children with congenital heart disease

**DOI:** 10.1186/s12871-023-02077-1

**Published:** 2023-04-19

**Authors:** Jiajia Huang, Daoqing Liu, Jie Bai, Hongbin Gu

**Affiliations:** 1 Department of Anesthesiology, Shanghai Children’s Medical Center, School of Medicine, Shanghai Jiao Tong University, Shanghai, P.R. China; 2 Department of Anesthesiology, Fujian Children’s Hospital (Fujian Branch of Shanghai Children’s Medical Center), College of Clinical Medicine for Obstetrics & Gynecology and Pediatrics, Fujian Medical University, Hengyu road 966, Fujian, 351114 P.R. China

**Keywords:** Congenital heart disease, Preoperative sedation, Median effective dose (ED_50_), Esketamine

## Abstract

**Background:**

Esketamine is commonly used as a premedication for its sedation effect. However, the proper dosage for intranasal use in children with congenital heart disease (CHD) has not been determined. This study aimed to estimate the median effective dose (ED_50_) of esketamine for intranasal premedication in children with CHD.

**Methods:**

Thirty-four children with CHD who needed premedication in March 2021 were enrolled. Intranasal esketamine was initiated at a dose of 1 mg/kg. Based on the outcome of sedation in the previous patient, the dose for the subsequent patient was either increased or reduced by 0.1 mg/kg, which was adjusted between each child. Successful sedation was defined as a Ramsay Sedation Scale score ≥ 3 and Parental Separation Anxiety Scale score ≤ 2. The required ED_50_ of esketamine was calculated using the modified sequential method. Non-invasive blood pressure, heart rate, saturation of peripheral oxygen, sedation onset time, and adverse reactions were recorded at 5 min intervals after drug administration.

**Results:**

The 34 children enrolled had a mean age of 22.5 ± 16.4 (4–54) months and a mean weight of 11.2 ± 3.6 (5.5–20.5) kg; American Society of Anesthesiologists classification I–III. The ED_50_ of intranasal S(+)-ketamine (esketamine) required for preoperative sedation in pediatric patients with CHD was 0.7 (95% confidence interval: 0.54–0.86) mg/kg, and the mean sedation onset time was 16.39 ± 7.24 min. No serious adverse events, such as respiratory distress, nausea, and vomiting were observed.

**Conclusions:**

The ED_50_ of intranasal esketamine was 0.7 mg/kg, which was safe and effective for preoperative sedation in pediatric patients with CHD.

**Trial registration:**

: The trial was registered in the Chinese Clinical Trial Registry Network (ChiCTR2100044551) on 24/03/2021.

## Background

Preoperative pediatric anxiety occurs in approximately 50–60% of children, especially those aged 1–5 years. This may have a negative psychological impact on children undergoing surgery [1, 2].

Preoperative sedation can alleviate perioperative anxiety of young children, thus creating a good condition for parental separation and anesthesia induction [3, 4]. Currently, various methods can be used to alleviate preoperative anxiety. In recent years large numbers of studies have focused on the effectiveness of non-pharmaceutical methods to achieve preoperative sedation in pediatric patients, although pharmaceutical sedation remains the general clinical practice.

Intranasal premedication is simple and minimally invasive compared with intramuscular or intravenous injections. Absorption is more complete, avoiding first-pass metabolism, and provides anesthesiologists with an alternative route for preoperative sedation. Midazolam, dexmedetomidine, and ketamine are sedatives for preoperative sedation in children, among which dexmedetomidine is more commonly used. However, dexmedetomidine will likely slow the heart rate [5–8]. The results of our previous study on preoperative medication in children with congenital heart disease (CHD) suggested that an intranasal drip of 2 mg/kg ketamine can be safely and effectively used for preoperative sedation in pediatric patients with CHD [9]. Esketamine is a new-type N-methyl-D-aspartate receptor inhibitor with the advantages of fast-acting and good sedative and analgesic effects; its sedative efficacy is 2-fold higher than that of ketamine, with minimal mental symptoms. In Europe, racemic ketamine has been replaced by its enantiomer S(+)-ketamine (esketamine), which has the advantage of anesthetic potency, minimal psychiatric adverse effects, little saliva secretion, fast action, and quick recovery [10–12].

Esketamine is also effective for pain relief because of ease of use and minimal effect on respiration. In addition, it has the feature of sympathetic activity, can increase systemic vascular resistance, and reduce intracardiac right-to-left shunting in children with cyanotic CHD, thus relieving hypoxia.

To the best of our knowledge, there is a lack of research on the median effective dose (ED_50_) of esketamine for intranasal premedication, especially in children with CHD, who are an exceptional cohort, and rational preoperative sedation can keep them in a quiet state and reduce vigorous hemodynamic fluctuations due to stress and anxiety.

Our pilot experiments suggested that a dose of 0.5–1.5 mg/kg could achieve an expected sedative effect in most children, and no evident inhibition of breathing and circulation was observed. Adverse reactions such as allergy, nausea, and vomiting have not been recorded. At present, the dosage of esketamine has not been clearly determined. To rationally apply esketamine to clinical practice, we designed this experiment to determine the ED_50_ of esketamine for intranasal premedication in children with CHD, and evaluated its safety, hoping that the results obtained in this study can provide useful references for the clinical application of the medication.

## Methods

### Participants

This prospective, randomized, single-blind study was approved by the ethics committee of by the Medical Ethics Committee of Shanghai Children’s Medical Center (SCMCIRB-K2020058-2) and registered in the Chinese Clinical Trial Registry Network (ChiCTR2100044551) on 24/03/2021. Inclusion of children with CHD was decided by the randomized number generated by computer simulation without sex limitation. Before signing the informed consent form, the guardians were informed of the possible risks associated with sedation and the precautions for care and monitoring.

Included in this prospective study were 34 children with CHD, aged from 4 to 54 months and weighing from 5.5 to 20.5 kg, who underwent elective surgery for CHD in March 2021. The exclusion criteria were: American Society of Anesthesiologists (ASA) classification ≥ IV; allergy to the drug to be tested; severe liver/kidney dysfunction; increased intracranial pressure; inability to separate from parents/guardians; history of intranasal surgery; front facial deformity; airway abnormality; and the presence of obstructive sleep apnea syndrome. According to the requirements for the research design of the sequential method, 6–8 crossover points were expected to be included [12], and the crossover point was defined as “the turning point for 2 adjacent cases from failure to achieve the expected effect” and “success in achieving the expected effect.“ Therefore, the inclusion of approximately 30 cases in the experiment would satisfy the statistical requirements.

#### Methods

Before surgery, the parents/guardians of the participants were advised to routinely fast their children for 6 h, avoid breastfeeding for 4 h and feed them with light food for 2 h. The patients and their guardians were escorted to the operating room 60 min before surgery. The patient’s preoperative status was assessed by the anesthesiologist in a single-blind manner, including ASA classification, heart rate monitoring, non-invasive blood pressure, saturation of peripheral oxygen (SpO_2_), and other baseline parameters. Esketamine (Jiangsu Hengrui Pharmaceuticals Co., Ltd., Lianyungang, China; batch no.: 200325BL) in a sterile hypodermic syringe (1 ml) was administered intranasally with the patient in the supine position in the guardian’s arms; the nasal mucosa absorbed it. Based on the pilot experiment results, the dose of esketamine was set as 1.0 mg/kg for the first patient, and the dose for the subsequent patient was determined by the extent of the expected effect achieved in the previous patient; if the expected effect was achieved, the dose gradient would be reduced, and if it failed to reach the expected effect, the dose gradient would be increased. Each nasal cavity was administered a half dose of esketamine, during which the child was in a supine position for 2 min and have the bilateral nasal wings pressed gently. Five min after drug administration, the patient’s heart rate and SpO_2_ were monitored. The depth of sedation was assessed by the Ramsay Sedation Scale score (Table 1), and at the same time, the nurse blinded to the study recorded the depth of sedation at the time of parental/guardian separation. In the present study, “success in reaching the expected effect” was defined using a ≥ 3 Ramsay Sedation Scale score 30 min after drug administration and a Parental Separation Anxiety Scale score (PSAS) (Table 2) ≤ 2 simultaneously. A dose gradient was defined as 0.1 mg/kg. The “effect onset time” was defined as the time from successful sedation to the time of reaching the Ramsay Sedation Scale score of ≥ 3. The sedation onset time and the occurrence of any adverse events such as nausea and vomiting, increased secretion, tachycardia or bradycardia, blood pressure abnormality, hypoxia, respiratory distress, and respiratory tract obstruction were recorded.

We confirmed that all methods were performed following the relevant guidelines and regulations.

### Research Parameters

The primary endpoint of the present study was the ED_50_ of esketamine for intranasal premedication in children with CHD. The secondary endpoints were the sedation onset time after drug administration and changes in respiratory and circulating functions at the designated observational time points.

### Statistical analysis

Statistical data were analyzed by SPSS 20.0 (IBM Corp., Armonk, NY, USA) and Excel Software (Microsoft Corporation, Redmond, WA, USA). The normality test of measurement data was performed using the Kolmogorov-Smirnov method, and data for normal distribution are expressed as the mean ± standard deviation. Under homogeneity of variance, a paired t-test was used for data analysis between groups. Measurement data for abnormal distribution or with uneven variance were tested using the Kruskal-Wallis method. P < 0.05 was considered statistically significant. Probit regression was used to calculate ED_50_ of esketamine and a 95% confidence interval (CI).

**Table 1:** Ramsay Sedation Scale

Definition	Score
Anxious and agitated or restless or both	1
Cooperative, oriented, and tranquil	2
Responds to commands only	3
Brisk response to a light glabellar tap or loud auditory stimulus	4
Sluggish response to light glabellar tap or loud auditory stimulus	5
No response to light glabellar tap or loud auditory stimulus	6

**Table 2:** Parental Separation Anxiety Scale(PSAS)

PSAS scores	PSAS 1 and 2: successful separation from the parents/guardians
1	easy to separate
2	sobbing but easy to cease
3	crying loudly and difficult to stop but without holding the parents/guardians and not letting them go
4	crying loudly and holding the parents/guardians and not willing to let them go

## Results

**Fig. 1:**
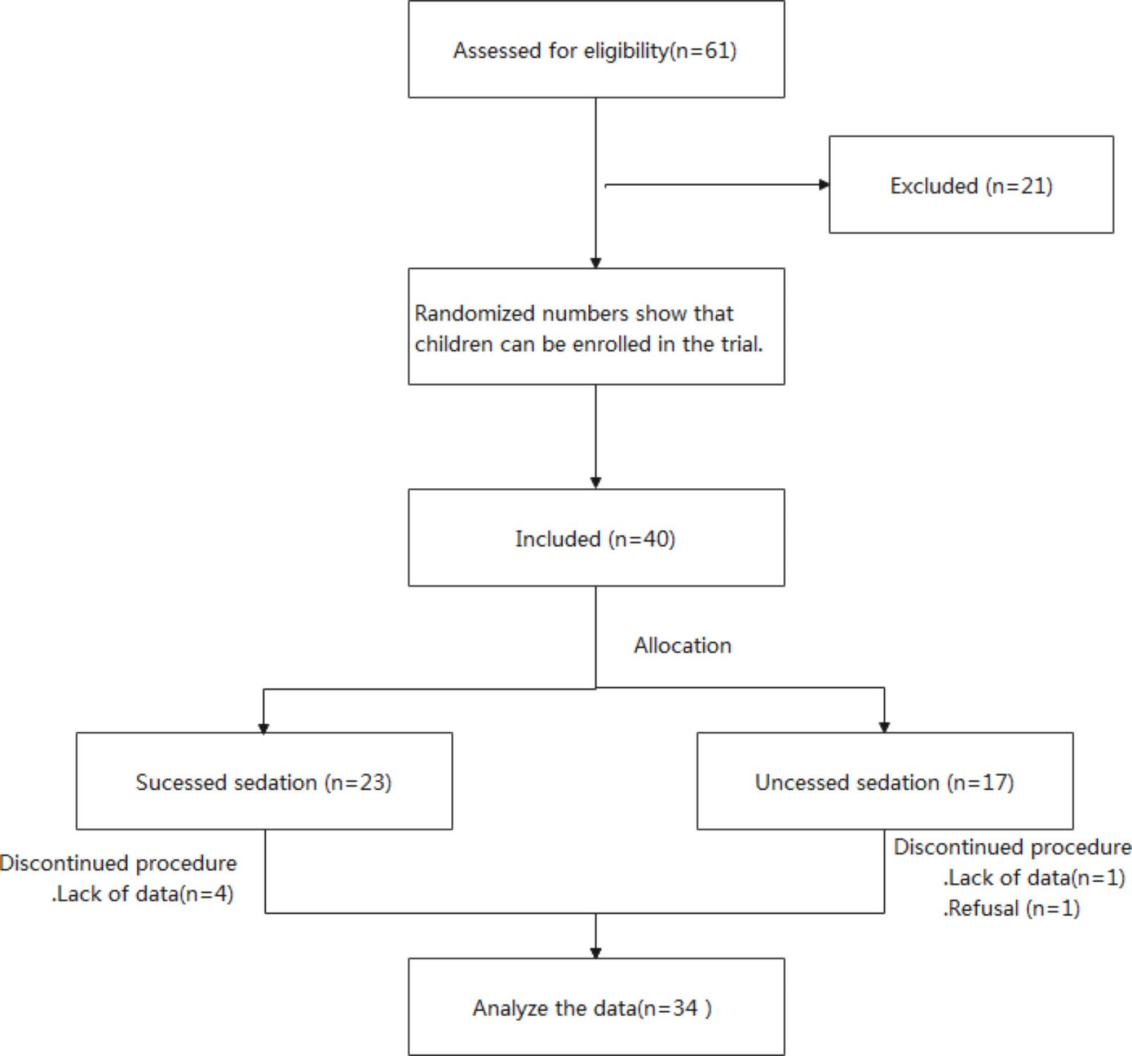
Participant flow chart Notes: Twenty-one patients were excluded from the study based on the exclusion criteria, 16 were excluded due to the inability to separate from parents/guardians, and 5 due to obstructive sleep apnea syndrome.

Thirty-four patients were included in the study,, ASAI–III,21 boys and 13 girls, with a mean age of 22.5 ± 16.4 (4–54) months and a mean weight of 11.2 ± 3.6 (5.5–20.5) kg. The mean weight for successful and failed sedation was 11.0 ± 4.0 kg and 11.5 ± 3.1 kg, respectively. The CHD of the patients included ventricular septal defect in 17, atrial septal defect in 9, pulmonary valve stenosis (PVS) in 2, Tetralogy of Fallot (TOF) in 2, and aortic stenosis in 1, patent ductus arteriosus in 1, and coronary right ventricular fistula in 1. The flow chart of the research process is indicated in Fig. 1.

**Fig. 2:**
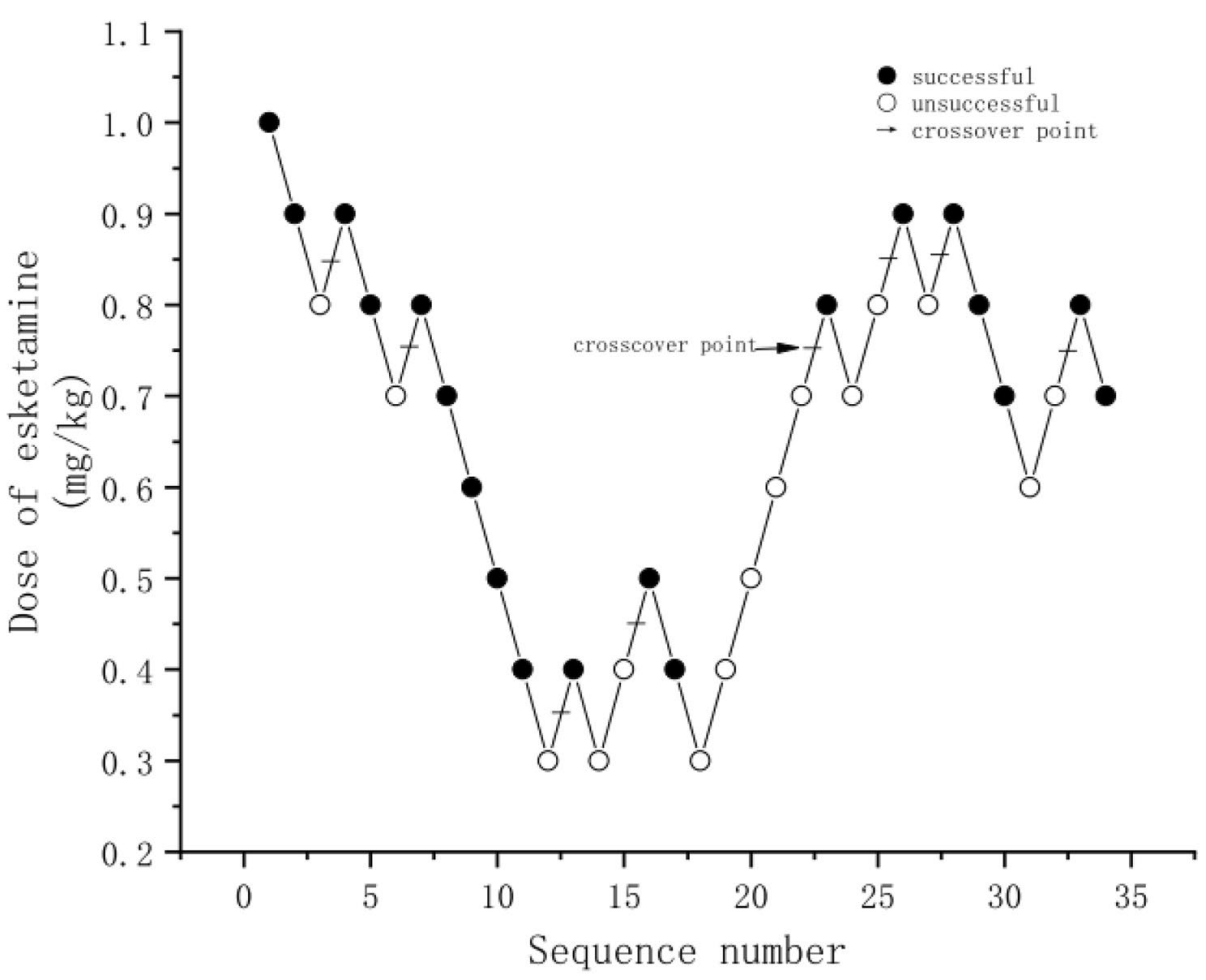
The Actual response curves and drug doses for intranasal esketamine Notes: The Esketamine dose for each patient. A successful dose is denoted by a solid circle; a failed dose is denoted by an open circle. The horizontal arrows represent crossover midpoints (failure to success).

Nineteen (55.9%) of the 34 patients who achieved successful sedation with intranasal esketamine were first included for analysis, in whom the effect onset time (min) was 20.5 ± 5.3 min, the quickest sedation onset time was 11.5 min, and the slowest was 28 min with a mean of 20.5 min. The other 15 patients who failed to achieve sedation were also included for analysis, including 9 cases of parental separation failure (4 children were unsuccessful in separating from their parents/guardians and sedation failure)and 6 cases of loss of sedation. After including the drug dose covariate and the dependent variable of response success, the optimal solution was obtained after 12 iterations. Using the probability regression method, the ED_50_ of esketamine was calculated as 0.7 (95% confidence interval [CI]: 0.54–0.86) mg/kg. Details of the actual response curves and drug doses of esketamine in the 2 groups are shown in Fig. 2.

Heart rate, SpO_2_, and mean arterial pressure at all designated time points were collected and analyzed for all patients (Table 3). No significant change was observed between the vital signs at baseline and those after drug administration. The Ramsay Sedation Scale scores obtained at the designated time points are shown in Table 4. The depth of sedation reached peak at 15–20 min after drug administration and remained till the end of the experiment. No significant adverse reactions were observed.

**Table 3:** Vital signs of the pediatric patients

Time	HR (bpm)	SpO_2_ (%)	MAP (mmHg)
0 min	124.4 ± 19.8	97.5 ± 2.5	81.3 ± 16.3
5 min	123.5 ± 20.1	97.8 ± 2.3	82.5 ± 19.3
10 min	119.5 ± 16.0	98.3 ± 1.9	79.4 ± 18.7
15 min	117.4 ± 12.9	97.9 ± 2.3	75.4 ± 12.4
20 min	117.6 ± 13.4	97.8 ± 2.2	82.4 ± 27.4
25 min	118.6 ± 15.6	97.9 ± 1.9	88.9 ± 19.5
30 min	118.5 ± 17.6	97.6 ± 2.4	84.3 ± 26.4

HR, heart rate; MAP, mean arterial pressure; SpO_2_, saturation of peripheral oxygen.

Note: The observational parameters were observed continuously. Analysis of the mean vital signs obtained at the designated time points indicated that the hemodynamics and other vital signs remained generally stable throughout the experimental period and showed no significant difference between successful and failed sedation.

**Table 4:** Ramsay Sedation Scale scores at all designated time points

Time	Ramsay score in all cases	Ramsay score in successful sedation cases
0 min	1.1 ± 0.3	1.2 ± 0.4
5 min	1.7 ± 0.8	1.9 ± 0.9
10 min	2.0 ± 0.8	2.3 ± 0.9*
15 min	2.4 ± 1.1	3.1 ± 1.1*
20 min	2.6 ± 1.3	3.5 ± 1.2*
25 min	2.7 ± 1.4	3.6 ± 1.2*
30 min	2.7 ± 1.4	3.7 ± 1.1*

Note: The Ramsay Sedation Scale scores in successful sedation cases were significantly higher than the mean values of the sedation scores in all included patients at all designated time points. The final Parental Separation Anxiety Scale at 30 min was 1.3 ± 0.5 in successful sedation cases, significantly lower than 1.8 ± 1.0 in the whole cohort. All the above results showed statistically significant differences. *, P < 0.05

## Discussion

We determined that the preoperative ED_50_ of esketamine was 0.7 (95% CI: 0.54–0.86) mg/kg for the pediatric patients in the series of this study; and no significant adverse events were observed throughout the experimental period. A 0.7 mg/kg dose was able to provide a safe and satisfactory effect against preoperative anxiety and an appropriate sedation effect in the children, as represented by the decreased anxiety, emotional stability, good cooperation, success in falling asleep, no bodily resistance, and smooth separation from their patients/guardians.

Midazolam, dexmedetomidine, and Ketamine are commonly used for preoperative sedation in children. An early in vitro animal experiment suggested that ketamine may be able to exert a potent diastolic effect on the intima of the pulmonary artery, thus reducing pulmonary circulation resistance [13, 14]. However, subsequent clinical studies demonstrated that the inductive dose of ketamine had no significant effect on the pulmonary arterial pressure of children with CHD and pulmonary vascular pressure of children with pulmonary hypertension [15, 16]. Esketamine is a new-type N-methyl-D-aspartate receptor inhibitor with the advantages of fasting action and evident sedative and analgesic effects. There have been many studies on esketamine administration for sedation and analgesia in children through an intravenous, intramuscular, or rectal route alone or in combination with other narcotics [17, 18]. Some studies reported that 2 mg/kg intranasal administration of esketamine can be absorbed quickly and effectively, reaching peak plasma concentration within a mean of 18 ± 13 min [19, 20]. Our study found Ramsay’s Sedation Scale score ≥ 3 occurred approximately 20.5 min in our cohort of children with CHD, which is consistent with the result reported in a previous study.

Unlike the previously reported studies, our study is one of the few studies that confirmed the ED_50_ within 95% CI after intranasal esketamine administration. We chose the modified sequential method to estimate the results because it is simple and requires a smaller sample size. Additionally, this method is suitable for obtaining quick results, and any effective dose can be determined using PROBIT regression analysis, such as ED90 and ED95. Many previous studies of oral or anorectal medication administration were concerned with pharmacodynamics and safety; and indicated 3–8 mg/kg as a maximum tolerable dose for oral medications. The common sedative effect of 1.5 mg/kg rectal administration was attributed to the high liver first-pass effect, produced after administration, and the bioavailability is only approximately 16%. Esketamine is relatively weak in young children, and its effect is not high as that of rectal administration of midazolam alone. In addition, the incidence of adverse effects is also relatively high [21] For this reason, some researchers have wondered whether it is possible to use larger doses and proposed to conduct further research on the relationship between pharmacodynamics and dosage. Considering that intranasal drug administration quickly acts on the brain due to bypassing the first-pass metabolism, high bioavailability, and loss of drug due to swallowing [22–24], esketamine is not added with any preservative. Therefore, it has little simulation on children, and the titer of esketamine is approximately 2-fold that of ketamine. In our pilot experiment, we selected 1 mg/kg of esketamine as the initial dose for intranasal premedication in our cohort of children with CHD to explore the ED_50_ and safety of intranasal esketamine.

Earlier studies on preoperative medication in children with CHD suggested that both intranasal administrations of 2 mg/kg ketamine and oral administration of 5 mg/kg ketamine were safe and effective for preoperative medication in children with TOF [9]. The sedation onset time of the intranasal medication group was faster than that of the oral medication group. The reason for the discrepancy of our ED_50_ value from other studies may be that: (1) the criteria for sedation assessment were different, we used the Ramsay Sedation Scale score ≥ 3 and PSAS ≥ 2 as the criteria for successful sedation; (2) we provided the parents/guardians of the participants with complete preoperative information and created a quiet preoperative waiting zone by separating them from other people unconcerned, and the waiting zone was equipped with cartoons and toys appropriate for the psychological features of children; and (3) we provided opportunities for family members to accompany the children and participate in their care. In addition few investigators had used intranasal administration for esketamine rather than ketamine for premedication in pediatrics, thus fewer results can be compared.

Our study found that the ED_50_ value of intranasal esketamine obtained in our patients was close to the result reported by Weber et al. [8]. Likewise, our patient cohort had no significant cardiovascular and respiratory adverse reactions, suggesting that an intranasal dose is not significantly different from a rectal dose. Weber et al. reported that compared with midazolam alone, combination use of 1–2 mg/kg esketamine could substantially increase the sedation score by 2.5 min after drug administration. Nausea and vomiting are common adverse reactions after intranasal administration of ketamine. The replacement of syringe drug administration by atomizer administration can reduce the incidence of nausea and vomiting by 50%. However we did not observe nausea and vomiting in our cohort of patients, possibly because the dose we used was relatively small and the observation time after sedation was relatively short.

Most researchers also define “cooperation without anxiety” as the criterion of successful sedation without considering the presence or absence of deep sleeping. Intranasal administration of 6 mg/kg ketamine created an appropriate depth of sedation in approximately 92% of cases, with a mean onset time of 5.79 ± 1.42 min. As the dose of ketamine is relatively large, some researchers recommended using the high-concentration of 100 mg/ml [25]. In our opinion, the main purpose of preoperative sedation is to relieve the anxiety of sick children and separate them from their parents peacefully. A deeper sleeping state not only requires a considerably larger amount of medication but will defer the resuscitation time for the children.

Although many studies believe that most children cannot comply with intranasal drug administration, only 1 child in our study had obvious non-compliance and resistance to intranasal premedication and needed physical restraint with the help of the parents for drug administration. The volume of drug administration in this study was less than 0.15 ml, and no child exhibited significant symptoms of pharmacochemical stimulation, suggesting that esketamine has minimal intranasal stimulation and the intranasal route is appropriate for drug administration. The intranasal stimulation symptoms observed in 36.1% of patients administered midazolam differed in several ways from those observed in this study [26], indicating that the pharmacochemical stimulation effect of midazolam is significantly higher than that of esketamine. Due to the concentration ratio of esketamine and data indicating a higher dose requires a higher volume for intranasal administration, which increases the discomfort of the patient and drug proportion passing through the throat and the amount absorbed by the gastrointestinal tract mucosa, we restrained the total amount of intranasal medication of esketamine to less than 0.3 ml, and the intranasal optimal volume dosage to less than 0.15 ml (the maximum amount for each nostril was 0.2 ml) to avoid oral administration of the excessive portion of the drug, which may have reduced bioavailability.

This study has several limitations. Firstly, we failed to make precise discriminations of the pediatric patient personality characteristics before the initiation of the study, knowing that different personalities may affect the sedative outcome on the day of surgery. In addition, we could not stratify the disease type and age because of the relatively small number of included patients. Therefore, we could not include patients of the same age group and the same kind of CHD for analysis. Thirdly, we did not use the atomizer. While atomizers offer a convenient means of administering nasal medication, their use is limited by the financial constraints faced by many caregivers who may require assistance in procuring the relatively expensive nasal atomizers. Finally, we did not further observe the sedative or analgesic effects after the patients entered the operating room.

## Conclusions

To our knowledge,this was the first study to report the ED50 of intranasal esketamine before cardiovascular surgery in young children with CHD. Our finding concerning the drug dosage differs from the results reported by most other studies, suggesting that drug administration should be implemented with caution and the target sedation protocol for children should be assessed individually. Other than the complex hemodynamic changes commonly induced by narcotics and sedatives, more attention should be paid to potential neurotoxicities arising from these agents and their interactions with subsequent anesthesia.

## Data Availability

The datasets used and/or analysed during the current study are available from the corresponding author on reasonable request.

## Abbreviations

ASA: American Society of Anesthesiologists
CHD: congenital heart disease
CI: confidence interval
ED_50_: effective dose
PSAS: Parental Separation Anxiety Scale
SpO_2_: saturation of peripheral oxygen
TOF: Tetralogy of Fallot
